# On Animal Charcoal as an Antidote

**Published:** 1848-09

**Authors:** B. Howard Rand

**Affiliations:** Philadelphia


### *On Animal Charcoal as an Antidote.* By B. Howard Rand, M. D., of Philadelphia.

The increasing number and fatality of accidents from the vegetable poisons and their active principles, of late years, has attracted the attention of the profession, for the purpose of seeking some antidote which might always be trusted, or some plan of treatment on which reliance might be placed.

M. Donné, some twenty years since, proposed the use of iodine, bromine, and chlorine, as antidotes, on the ground that they combine with the poisonous principle and form an inert compound. It has been found, however, that this inert compound is readily decomposed by acids, the poison being liberated unchanged; but a more serious objection to the use of these elements as antidotes is, that they are themselves among the irritant poisons, and must be given with caution.

Tannic acid has also been proposed, but the fact of its compound with the poisonous principle being slightly soluble in water, and freely so in acetic acid, would seem to render it ineligible. M. Bussey,* has recently suggested the use of magnesia, but the experiments with it appear to have been confined to the mineral poisons alone.

The usual mode of procedure, then, in such cases, and the only one on which any hopes of success were founded, has consisted in the free evacuation of the stomach, and the use of such general measures as in each case might seem to be indicated.

In the Transactions of the Medical Society of London, new series, Vol. 1,† is a paper by Dr. A. B. Garrod, detailing some experiments in which he employed purified animal charcoal as an antidote. This is prepared from ivory black, by digesting it in dilute chlorohydric acid to remove the earthy matters, afterwards washing and heating it to redness in a covered crucible. The subjects of his experiments were dogs, rabbits, and guinea pigs. He administered large doses of opium, belladonna, aconite, nux vomica, delphinium, stavesacre, white hellebore, and their alkaloids, as well as digitalis, hemlock, tobacco, elaterium, ipecac-

* Gazette Medicale de Paris, 23d March, 1846.
† Condensed in Braithwaite's Retrospect, Vol. xiii.

uanha, hydrocyanic acid, cantharides, and arsenious acid, without injurious consequences, when sufficient animal charcoal was given simultaneously with them, or, in some cases, before the peculiar effects of the drug were developed. It also prevented, but less completely, the action of the bichloride of mercury and of the salts of copper and lead.

Dr. Garrod concludes from his experiments :—

1st. That animal charcoal has the power of combining, in the stomach, with the poisonous principles of animal and vegetable substances, and that the compounds thus produced are innoxious ; therefore, when given before these poisons have become absorbed, it will act as an antidote.

2d. That animal charcoal will absorb some mineral substances, and render them inert ; but so large a quantity of the charcoal is required, that it is not so well adapted for many poisons of this class, as their own special antidotes ; the effects of arsenic, however, appear to be better combated by this than by any other article.

3d. That a certain *amount* of animal charcoal is required, about half an ounce to each grain of morphia, strychnia, or any other alkaloid—but, of course, much less for the substances from which they are obtained,—as opium, nux vomica, &c.; a scruple of nux vomica not requiring more than half an ounce of charcoal.

4th. That the antidote itself exerts no injurious action on the body.

Dr. Garrod also remarks that, " Ivory black has a certain amount of antidotal power, but would be required in very much larger quantities, containing above 90 per cent of earthy matter. Vegetable charcoal possesses but a small antidotal power compared with animal charcoal. Lamp black is totally devoid of the property." He also suggests its use in poisoned wounds, syphilis, &c., in the form of a poultice, to absorb the virus and prevent its being taken into the system.

Previous to the publication of Dr. Garrod's paper, the researches of MM. Wapen, Holpoff, Warrington, and others, had proved that animal charcoal is capable of removing from their solutions—in some cases only by the aid of heat—the bitter, resinous, or active principles of quassia and the other simple bitters ; of colocynth, aloes, and other purgatives ; of krameria and the other astringents; of guaiacum, cinchona, opium, nux vomica, and, in short, of all vegetable substances submitted to its influence.

MM. Wapen, Chevalier, and Graham, have also discovered that it possesses the property of precipitating the oxides of a large number of metals from their solutions. Among the salts decomposed, may be mentioned the bichloride and the nitrates of the protoxide and deutoxide of mercury, the tartrate of antimony and potassa, nitrate of silver and of cobalt, neutral acetate of lead, chloride of tin, sulphates of copper, zinc, and protoxide of iron. The acid salts, and many of the neutral salts, were exempt from its action ; among these were the arsenites of potassa and soda, the acid nitrate of mercury, and the cyanide and ferrocyanide of potassium. It precipitates the antimonic, tungstic, and plumbic acids, *but has no action on arsenious acid.* Iodine and the ioduretted iodide of potassium, were removed from their solutions, but sulphur was not.

To Dr. Garrod the credit is due of having first applied these facts to toxicology. It is true, however, that M. Bertrand, in 1813, proposed powdered charcoal as an antidote to arsenic, and is reported to have swallowed five grains of the drug in an emulsion of charcoal without bad effect. It is not stated, however, that he used *animal* charcoal, and its claims as an antidote, strictly speaking, to arsenic, have since been disproved.*

The method of preparing pure animal charcoal from ivory black, before mentioned, is tedious and wasteful, only ten per cent being obtained, while the amount of acid used is considerable. A very good and pure charcoal is obtained by calcining leather scraps or blood with pearlash, washing and re-heating in a close crucible ; it was this kind of charcoal which was principally used in the experiments to be detailed. Much of the discrepancy of experimenters with this substance, is undoubtedly due to their use of an impure specimen of it.

Taken internally, in moderate quantity, pure animal charcoal causes a sensation of warmth in the epigastric region, which soon subsides ; the sensation produced by the hard particles in their passage through the pharynx and œsophagus, is disagreeable, and sometimes very persistent. In large doses, or when the stomach

* Taylor on Poisons. Amer. Ed. p. 76.

is irritable, it produces vomiting, and sometimes considerable evidences of gastric irritation.

With a view of ascertaining more accurately the value of the proposed antidote, the writer performed a number of experiments, from which the following are selected as having the most direct bearing upon the subject.

1. One grain of pure morphia was swallowed with about an ounce of pure animal charcoal, in warm water; no narcotic symptoms supervened, but there was some gastric irritation which subsided in the course of the day.

2. One grain of sulphate of morphia was digested with pure animal charcoal until all bitterness was removed; the liquid, filtered off and swallowed, produced no effect.

3. Ten grains of extract of belladonna were swallowed with two drachms of the charcoal; there followed vertigo, dilated pupils, dimness of vision, exceeding dryness of the throat, and desire to sleep, all of which symptoms were relieved by the spontaneous vomiting of a very acid matter and the use of stimuli. The pupil remained dilated through part of the next day.

4. The last experiment was repeated, an antacid having been premised, and the proportion of charcoal being doubled. Some dryness of the throat followed, but with no other symptom of the influence of the drug.

5. Fifteen grains of powdered digitalis were taken, with three drachms of the animal charcoal, without the slightest disturbance of the functions.

6. Twelve drops of the officinal hydrocyanic acid were swallowed, with two drams of the pure charcoal, without a sedative result.

7. One grain of strychnia, dissolved by the aid of a drop of chlorohydric acid, was digested with animal charcoal until all bitterness was removed. The solution filtered and swallowed produced no effect; a similar solution evaporated and tested with nitric acid gave no red tinge.

8. One grain of strychnia was swallowed, with an ounce of pure animal charcoal; no effects due to the strychnia could be perceived.

9. The purgative extracts were next tried, but produced no effect when sufficient animal charcoal was taken.

10. Camphor and musk were removed by animal charcoal from their tinctures, so far that they did not precipitate on the addition of water.

11. Phosphorus was removed from its ethereal solution by the charcoal.

12. Iodine was so far removed from its tincture and compound solution as not to strike a blue colour with starch, and the iodine could not be liberated from the animal charcoal at a red heat.

13. Arsenious acid and a solution of arsenite of potassa were apparently unaffected, either in the hot or cold solution, by animal charcoal. This result, although it agrees with those of MM. Wapen and Graham, does not with the observations of Dr. Garrod, who states that animal charcoal "has greater power of removing arsenic from its solution than the hydrated sesqui-oxide of iron."

14. A solution of the bichloride of mercury, being treated with animal charcoal, gave, on filtration, no precipitate with ammonia.

It has been stated that the action of this agent is purely mechanical, enveloping the poison, thus protecting the coats of the stomach from its action, and preventing absorption, and that if given *after* the poison has been swallowed, instead of with it, it would prove useless. It has been said, also, that, considering this to be its mode of action, any ordinary inert powder, would answer equally well. This may be true as regards arsenic, which is not removed from its solutions by animal charcoal, and such is the result of the experiments of Orfila and Christison; but, the case of the alkaloids is different—they are wholly removed from their solutions by the animal charcoal entering into a firm union with them. Dr. Garrod's experiments show that vegetable charcoal and lamp black are nearly or quite useless in counteracting the effects of poisons, and that in the milder poisons the administration of the proper article might be delayed ten or fifteen minutes with safety. These facts certainly seem to show that it acts otherwise than by merely enveloping the poison; but, granting such to be its action, if it only prevents the fatal effects of the poison, we have all that is desired. Experience alone can determine its positive value as an antidote, properly so-called.

When given as an antidote, the animal charcoal should be mixed with water as hot as the patient can swallow, as its action is much aided by an elevated temperature; the administration of large quantities of this agent alone (tepid) will often provoke emesis, which should be promoted by emetics or the stomach pump. Should a sufficient quantity of the poison have been absorbed to produce its peculiar effects, they are to be combated by general measures. It would be proper in many cases to combine the charcoal with an antacid, as any considerable quantity of free acid in the stomach is found to interfere with its action.

Since pure animal charcoal requires time and care in its preparation, and does not deteriorate by keeping, suitable quantities should be kept on hand by apothecaries, and by physicians in the country. It was found, however, upon enquiry of numerous apothecaries in this city, that many did not keep the article, and of those who did, few had more than two or three ounces, and that sometimes of questionable purity.

In conclusion:—we are perhaps justified in drawing, from the present state of our knowledge on this subject, the following conclusions:

1st. That animal charcoal has the power of withdrawing, when used at a proper temperature and in sufficient quantity, most, if not all, known vegetable and animal poisonous principles, and certain mineral poisons from their solutions.

2d. That, given at the same time, with, or shortly after these poisons have been swallowed, it prevents their deleterious action.

3d. That, given in cases of poisoning, it can exert no injurious influence, but, on the other hand, promotes vomiting, entangles the poison and protects the coats of the stomach against it.

4th. That, although it cannot be substituted for the usual antidotes in poisoning by mineral substances, yet it may be usefully employed in conjunction with them or in their absence.

The necessarily confined space of this paper, has compelled the writer to pass lightly over many important facts, and to leave untouched some points of interest, but of minor importance; among these are the questions of the possibility of the antidote being administered in time to be of use, in poisoning by the more rapidly fatal agents; of its probable modus operandi, and that of the loss necessarily resulting in filtering the *warm* solution of the alkaloids, during their preparation, through animal charcoal, in order to decolorize them.

